# Epidemiology of Injuries in Belgium: Contribution of Hospital Data for Surveillance

**DOI:** 10.1155/2014/237486

**Published:** 2014-04-28

**Authors:** Christelle Senterre, Alain Levêque, Lionel Di Pierdomenico, Michèle Dramaix-Wilmet, Magali Pirson

**Affiliations:** ^1^Research Center of Epidemiology, Biostatistics and Clinical Research, School of Public Health, Université Libre de Bruxelles, Route de Lennik 808, CP 598, 1070 Brussels, Belgium; ^2^Research Center of Health Economics, Hospital's Management and Nursing, School of Public Health, Université Libre de Bruxelles, Route de Lennik 808, CP 592, 1070 Brussels, Belgium

## Abstract

*Objectives*. Investigating injuries in terms of occurrences and patient and hospital stay characteristics. *Methods*. 17370 stays, with at least one E code, were investigated based on data from 13 Belgian hospitals. Pearson's chi-square and Kruskal-Wallis tests were used to assess the variations between distributions of the investigated factors according to the injury's types. *Results*. Major injuries were accidental falls, transport injuries, and self-inflicted injuries. There were more men in the transport injuries group and the accidental falls group was older. For the transport injuries, there were more arrivals with the support of a mobile intensive care unit and/or a paramedic intervention team and a general practitioner was more implicated for the accidental falls. In three-quarters of cases, it was a primary diagnostic related to injury and poisoning which was made. The median length of stay was nearly equal to one week and for accidental falls, this value is three times higher. The median cost, from the social security point of view, for all injuries was equal to €1377 and there was a higher median cost within the falls group. *Conclusion*. This study based on hospitals data provides important information both on factors associated with and on hospital costs generated by injuries.

## 1. Introduction

In Belgium, as in other countries all over the world, injuries remain a public health problem. In 2009, in the European Union, mortality due to external causes was equal to 30 deaths for 100000 inhabitants, with higher values in Belgium than in its neighboring countries: 36.8/100000 versus 31.0/100000 in France, 29.9/100000 in Luxembourg, 19.8/100000 in Germany, and 16.4/100000 in the Netherlands [1]. Still in 2009, without considering the age groups, suicide and transport injuries were among the top ten causes of death in Belgium, with a proportionality mortality ratio (PMR) of 1.9% (7th place in the ranking) and 1.0% (10th place in the ranking), respectively. In the 15–24-year age group, suicide and transport injuries were the two first leading causes of death, with a PMR of 36.7% and 22.9%, respectively. The situation was nearly the same in the 25–44-year age group (suicide PMR equal to 22.9%, 1st place in the ranking, and 11.6% for the transport PMR, 3rd place in the ranking) [2]. Next to this high mortality, there is significant morbidity and also an important burden of these injuries. In the European region, according to the Global Burden of Disease report road traffic injuries are in the 6th place in the ranking of the leading causes of burden diseases with 3.7 million of disability-adjusted life year (DALY's), corresponding to 2.4% of total DALY's; self-inflicted injuries are in the 10th place with 3.1 million DALY's, corresponding to 2.0% percent of total DALY's [3]. Generally, mortality is well documented, especially for certain type of injuries (e.g., traffic injuries), but there is lack of information about the morbidity. However, several types of data sources are available for injury morbidity surveillance, for example, population based survey data and emergency department data or hospital data [4]. Within the European region, injuries represent an estimated number of 7200000 hospital admissions, 34800000 emergency department attendances, and 18600000 other medical treatments [5]. According to the summary of injury statistics for the years 2008–2010 published by the European Association for Injury Prevention and Safety Promotion (EuroSafe) [6], in Belgium, the estimated percentage of injury related to hospital discharge is equal to 10%. Although hospitalized injuries represent a small proportion of nonfatal injuries, they are generally more severe and are associated with higher medical and treatment cost than those who are not treated in hospital, and because patients admitted to hospital usually have longer stays than those treated in the emergency department, hospital inpatient records usually contain more detailed and accurate information about the diagnosis of injury than emergency department visit records [4, 7, 8].

In Belgium, the Minimal Clinical Dataset is a standardized and concise summary of the patient's medical record that general hospitals are required to register since 1990. The registration has the objectives to identify needs for hospital equipment, to define the standards of qualitative and quantitative recognition of hospitals and their services, to organize the funding of hospitals, to determine policy on the exercise of the art of healing, and to define epidemiological policy [9]. In the early 2000s, a published ministerial circular has strongly encouraged the registration of the codes E [10]. To our knowledge, no epidemiological studies have investigated specifically the distribution of E codes in the database, nor patient characteristics, and even less the cost associated with the hospitalization. So the objectives of our study were to investigate these E codes, based on the more available recent data, in terms of occurrences, patient characteristics, and hospital stay characteristics.

## 2. Methods

### 2.1. Cases' Selection

**Figure 1:**
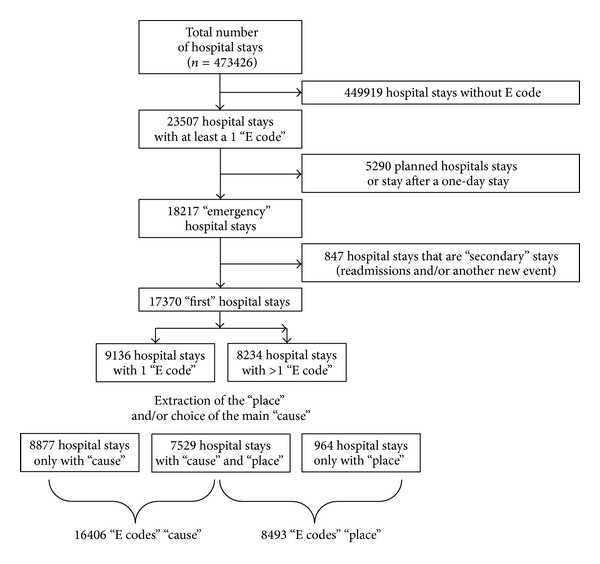
Flow chart of the selection of the stays.

These analyses were performed with the 2010 data from 13 Belgian hospitals, which are included in the PACHA project, a project focused on the analysis of stays and pathologies cost. These hospitals are either private or public. In 2010, the total of inpatient stays from this sample represented 11.4% of all Belgian inpatient stays. On the 473426 available stays (inpatient stays and day care stays), only hospital stays with at least one external cause (E codes) were selected. There were up to 3 E codes for some case files (Figure 1). Data were coded according to the ninth revision of the International Classification of Diseases, clinical modification (ICD-9-CM), so the E codes' groups taken into account in this study were presented in Table 1 [11]. As in the EUROCOST project [12, 13]—concerning the cost estimation of injury-related hospital admissions in European countries—and as in Meerding and colleagues [14] study on the costs of injuries in the Netherlands, we have not considered the following E codes: “misadventures to patients during surgical and medical care” [E870–E876], “surgical and medical procedures as the cause of abnormal reaction of patient or later complication, without mention of misadventure at the time of procedure” [E878-E879], and “drugs and medicinal and biological substances causing adverse effects in therapeutic use” [E930–E949].

In the ICD-9-CM, causes and place of occurrence were found in the same chapter, so we have first differentiated, on one hand, the causes and, on the other hand, the places of injuries. Because of this absence of differentiation between place and cause, all the patients have not both code for the cause and another one for the place (Figure 1). Besides that, for the management of the stays with more than one cause we have created, for the extraction of a “main” external cause, a decision algorithm based on the gravity's perception. For example, if a patient had both a code for an accidental fall and another one, like transport injury [E800–E848] or accidental poisoning by drugs, medicinal substances, and biologicals [E850–E858] or suicide and self-inflicted injury [E950–E959] or another homicide and injury purposely inflicted by other persons [E960–E969], we have taken into account those second codes. In other words, if a person attempts suicide with drugs and falls after having ingested the drugs, it is more important to consider the suicide attempt than the fall.

### 2.2. Associated Factors

The other variables taken into account have been chosen to try to follow the patient from its arrival to its discharge. The demographic characteristics of the patients were gender and age and we also have information about the moment (month and day), the type and the initiative of the hospital's admissions, the primary diagnosis that justifies the hospitalization, the stays description (in terms of care units), the type of discharge, the patient's destination, the cause of death, the type and the length of stay, and finally the cost incurred from the social security point of view.

#### 2.2.1. Type of Admission

Based on the preexisting categories, we have regrouped the type of admissions in 4 categories that may already reflect certain gravity of the injury: 1: admissions through the emergency department (ED) without ambulance; 2: admissions through the ED with an ambulance; 3: admissions through the ED with an ambulance and also with the intervention of a MICU, mobile intensive care unit, and/or a PIT, paramedic intervention unit; and finally 4: unplanned admission, meaning emergency hospitalization not through the emergency department.

#### 2.2.2. Primary Diagnosis

It is defined as the affection which, upon medical examination, proved to be the main cause of patient's admission. For the description of this primary diagnosis, we have used the chapter headings of the ICD-9-CM classification (Table 2) and we have detailed more the chapter concerning the injury and poisoning (Table 3).

#### 2.2.3. Stays Description

The different services were the emergency unit, the one-day unit, the intensive care unit, the burn unit, the diagnostic and surgical care unit, the diagnostic and medical care unit, the geriatric unit, the pediatric unit, the neuropsychiatric unit, the specialized unit for the treatment and rehabilitation (with cardiopulmonary affections, musculoskeletal affections, neurological diseases, palliative care, polychronic diseases, and psychogeriatric affections), and finally the obstetric and neonatal unit.

During the stays, patients could change, one or more times, service according to the evolution of their health status. We therefore have investigated up to three consecutive services, with a focus on the passages by the intensive care unit or by the specialized units for the treatment and rehabilitation. Focus was made because a stay in one of these services may reflect certain gravity of the health status.

#### 2.2.4. Type of Discharge, Patient's Destination, and Cause of Death

Based on the preexisting categories, we have regrouped the type of discharge in 5 categories. Two categories were related to the hospital's discharge with or without medical advice and the 3 others were related to a transfer to another place: 1: for specialized care, 2: for reeducation, or 3: for logistic reasons (e.g., for financial problems). The patient's destination was taken into account because this variable contains relevant information in terms of patient's redirecting to psychiatric homes/hospitals or to elderly homes. The other variable's categories were the domicile, another hospital, or all other possible destinations such as jail or a boarding school. By the way of these two variables it was also possible to know if the patient was dead during their stay. The cause of death was reported in one of the fields of the patient file. It is important to note that the reported cause of death was the one that appears on the death certificate and that this cause is, for the nonnatural deaths, the circumstances of the accident (or the place of injuries if no type of injuries was reported) unless there was a morbid condition underlying (e.g., the neoplasms and the circulatory diagnoses). This death can be natural or nonnatural, so for this variable, there was coexistence of codes related to diseases and codes related to external causes (Tables 2 and 3).

#### 2.2.5. Type, Length, and Cost of Stays

The type of stays can provide first information about the gravity, because a patient who only stays for a day hospitalization is probably less affected than a patient who stays as an inpatient. Furthermore, patients in the case of the day hospitalization, day surgery, or outpatient emergency have a length of stay equal to one day, so they are not taken into account when it is the question of the estimation of mean or median length of stay (reported in days). The estimation of the costs borne by social security (reported in euros) was also only based on the inpatient stays and the costs were broken down into costs resulting from medical procedures, from pharmaceutical products, and from day lump sums. The percentage of each of these 3 specific costs in relation to the whole cost was also calculated.

### 2.3. Statistical Analyses

Categorical variables were described with both absolute and relative frequencies and the variations between these distributions according to the different groups of injuries were assessed using Pearson's chi-square test in accordance with the Cochran guidelines cited by Altman [15] (80% of the cells in the table should have expected frequencies greater than 5 and all cells should have expected frequencies greater than 1). Because of the skewness of the quantitative variables, the median and the 25th and the 75th percentiles were reported and the Kruskal-Wallis nonparametric test was used to assess the variation between the distributions of these variables according to the different groups of injuries. The significance level for all tests was 0.05 and all statistical analyses were performed using Stata/SE 12.0 for Windows (TX: StataCorp LP).

## 3. Results

### 3.1. E Codes Observed

Table 4 shows that the three major groups of injuries observed were accidental falls [E880–E888] (58.2%), transport injuries [E800–E848] (11.2%), and suicide and self-inflicted injuries [E950–E959] (10.6%). According to these observations the next results would be presented on one hand for all stays and on the other hand for these three groups. Concerning the place of occurrence, there was obviously a correlation with the causes: home accidents [E849.0] (42.1%), accidents occurring in a residential institution [E849.7] (14.9%), and street and highway accidents [E849.5] (13.4%) were the major places observed.

### 3.2. Demographic Characteristics of the Patients

There were significantly more men in the transport injuries group than in the two other groups (66.7% versus 39.3% and 37.0%, resp.) and the patients in the accidental falls were older than those in the two other groups (median age equal to 74 years versus 32 years and 41 years, resp.) (Table 5).

### 3.3. Moment, Type, and Initiative of the Hospital's Admissions

**Figure 2:**
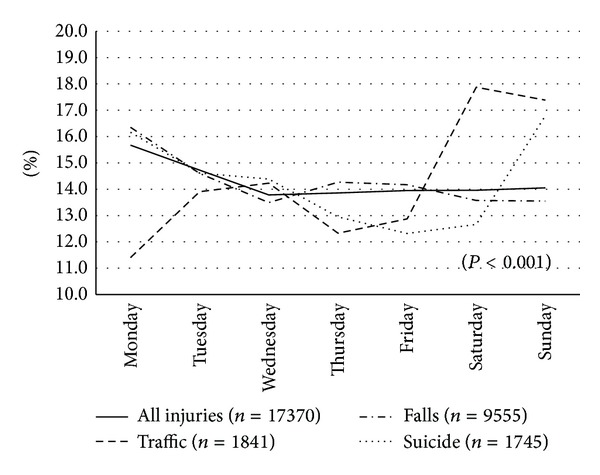
Variation of the proportions of injuries according to the weekdays.

**Figure 3:**
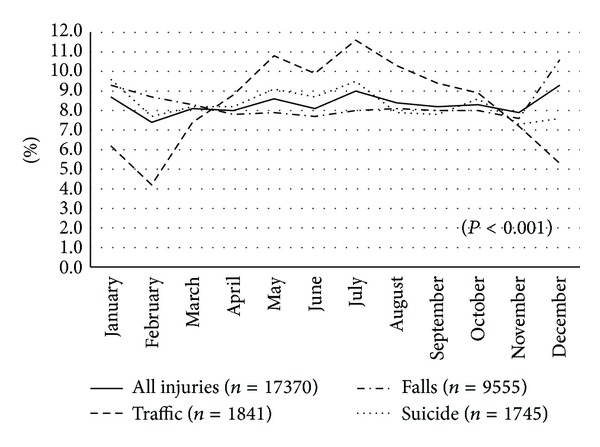
Variation of the proportions of injuries according to the months of the year.

Figures 2 and 3 show the repartition of all injuries according to the weekday and month of the year. For all injuries, there were more admissions on Mondays (15.7%) and Tuesdays (14.7%) than on other weekdays (from 13.8% on Wednesdays to 14.1% on Sundays). The same trend was roughly observed for the falls, but there were significantly (*P* < 0.001) more admissions for traffic injuries on Saturdays and Sundays (17.9% and 17.4%) and more admissions for suicide and self-inflicted injuries on Sundays (16.8%) and Mondays (16.2%). For the month of admissions, the percentages were nearly the same for each month (from 7.4% in February to 9.3% in December) for all injuries, but the repartition was significantly (*P* < 0.001) different between traffic injuries, accidental falls, and self-inflicted injuries. The highest proportions of admissions for traffic injuries were observed between May (10.8%) and August (10.3%). For accidental falls, December (10.6%) was the most observed, and finally for self-inflicted injuries, higher proportions were observed in January (9.6%) and July (9.5%).

Regarding the type of admission, there were significantly (*P* < 0.001) more arrivals through the emergency department with the support of a mobile intensive care unit and/or a paramedic intervention team in the group of the transport injuries than in the accidental falls and suicide groups (36.4% versus 10.3% and 23.5%, resp.). Relating to the person at the initiative of the admission, the general practitioner was more implicated in the case of an accidental fall (21.9% versus 2.5% and 7.0%, resp.), while a third party was implicated in nearly half the situations of transport injuries and a little more than forty percent of the self-inflicted injuries (47.4% and 43.3% versus 27.9%, resp.). It was also observed that a not inconsiderable proportion of admissions were due to the initiative of the patient (Table 6).

### 3.4. Primary Diagnosis That Justifies the Hospitalization

Table 7 showed that for all injuries, in three-quarters of cases (74.6%), it was a diagnostic related to injury and poisoning [800–999] which was made, and this proportion was significantly different (*P* < 0.001) between the three studied groups, with a lower proportion in the accidents' falls group relative to the transport injuries and the self-inflicted injuries (72.2% versus 93.1% and 90.0%, resp.). In the accidental falls group, there were diseases of the circulatory system and in the self-inflicted injuries group mental disorders were found in a higher proportion than in the other groups. If we look in more detail to the injury and poisoning diagnosis group, half of it consisted of fractures: 22.8% for fractures of the lower limbs [820–829], 16.6% for fractures of the upper limbs [810–819], 8.2% for fractures of the neck and the trunk [805–809], and finally 3.3% from fracture of the skull. In the transport injuries group, besides the nearly fifty percent of fractures (17.7% for the neck and trunk, 17.1% for the upper limbs, and 14.4% for the lower limbs), there were almost fifth (18.1%) of intracranial injuries [850–854]. In the accidental falls, seven cases of ten were also fractures: 36.1% for the lower limbs, 22.9% for the upper limbs, and 10.2% for the neck and the trunk. Finally, in the self-inflicted injuries group, ninety-one percent were poisoning by drugs and medicinal and biological substances [960–979] accounting for 84.1% and 6.9% were toxic effects of substances chiefly nonmedicinal as a source [980–989] (Table 8).

### 3.5. Stays Description

**Figure 4:**
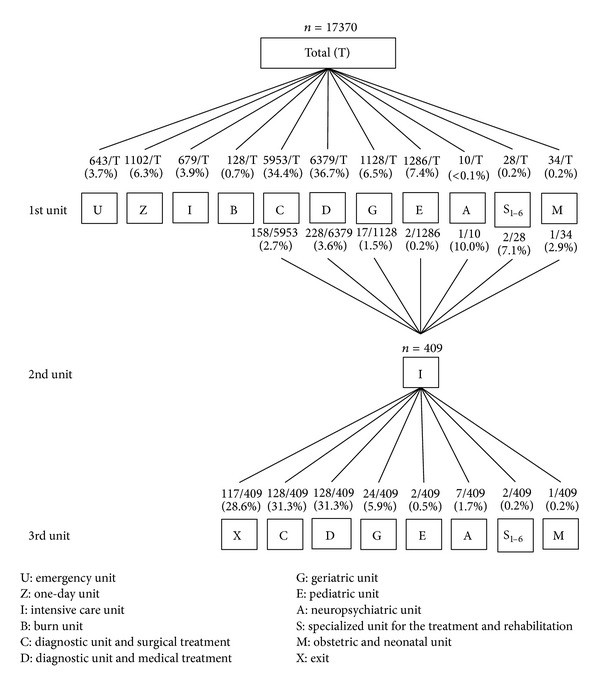
Stays description with a focus on the intensive care unit as the second unit.

**Figure 5:**
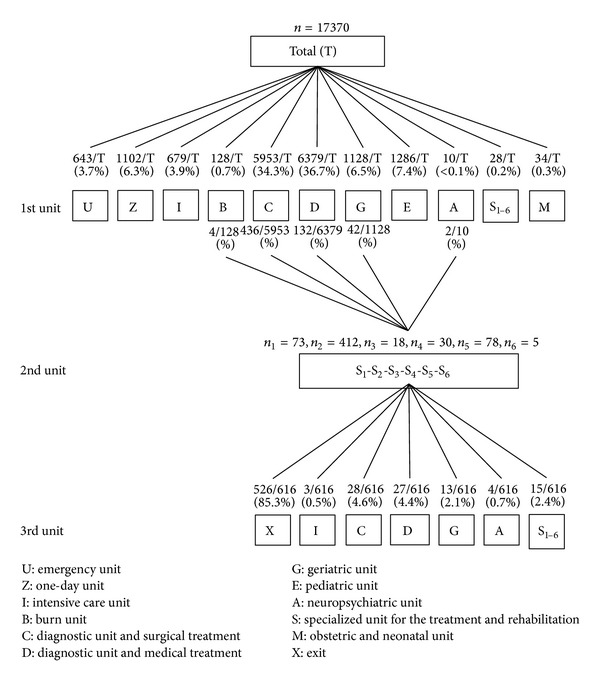
Stays description with a focus on the specialized unit for the treatment and the rehabilitation as the second unit.

Figure 4 shows that two-thirds of the 17370 patients have stayed in a diagnostic and surgical care unit (C) (34.4%) or in a diagnostic and medical care unit (D) (36.7%) on their arrivals, and a little less than five percent (3.9%) were directly admitted to the intensive care unit. For 409 of the 17370 patients, the stay was extended by a passage in the intensive care unit. Therefore, 6% of all patients remained at least one day in an intensive care unit. After the stay in the intensive care unit, 28.6% patients have left the hospital. Among the patients who were returned to one other service, after the stay in the intensive care unit, 31.3% were returned to a C unit and 31.3% other were returned to a D unit.  Figure 5 shows that, regarding the specialized units for the treatment and the rehabilitation, 616 patients have gone through these units after a first ward, with 412 for the musculoskeletal affections. Finally, 85.3% percent of these 616 patients were discharged from the hospital after their stays in one of the six specialized units.

### 3.6. Type of Discharge, Patient's Destination, and Cause of Death

Seven hundred and twenty-six patients of the entire sample have lost life at hospital, with more than half of the deaths (*n* = 490, 67.5%) concerning patients admitted for accidental falls [E880–E888]. We have also observed 50 deaths (6.9%) concerning patients admitted for accidents caused by submersion, suffocation, and foreign bodies [E910–E915], 46 deaths (6.3%) concerning patients admitted for transport injuries [E800–E848], and 22 deaths (3.0%) concerning patients admitted for suicide and self-inflicted injuries [E950–E959]. Sixty-eight deaths are related to patients for whom we only have the place of occurrence, and the two major places reported were the residential institution [E849.7] for 45 cases (6.2%) and the home for 17 cases of death (2.3%) (data not shown). Therefore, the proportion of death was significantly higher in the group of accidental falls than in the other two groups (5.2% versus 2.5% and 1.3%, resp.) (Table 9).

About the type of discharge, the proportion of transfer for specialized care was higher in the self-inflicted group (5.8% versus 3.6% and 2.4%, resp.) and the proportion of discharge without medical consent was also higher in this group than the other two (8.0% versus 1.2% and 1.0%, resp.). Concerning the destination after the discharge, a large part of the patients returned to their home, but in the accidental falls group nearly 14% went to elderly homes and in the self-inflicted injuries groups 5.7% went to psychiatric structures (Table 9).

If we look in more detail to the causes of death, we observe that 31.6% in the transport group, 17.5% in the accidental falls group, and 29.4% in the self-inflicted injuries group were directly related to the type of injuries which have led to the admission, and for another part it was the injury and poisoning cause which has been mentioned (23.7%, 12.5%, and 41.2% in the three groups, resp.). The other major cause of death observed was a disease of the circulatory system both for the transport injuries group and for the accidental falls group (36.8% and 27.6%, resp.) (Table 10).

### 3.7. Type, Length of Stay, and Cost Incurred

Concerning the hospital stays, the inpatient stays were the most observed. According to the three groups investigated the proportion of inpatients was significantly higher in the suicide group (94.7% versus 91.3% and 92.4%, resp.). Overall, the median length of stay was nearly equal to one week (6 days) and in the accidental falls group, this value was at least three times higher than in the two other groups (9 days versus 3 days and 2 days, resp.). The median cost borne by social security for all injuries was equal to €1376.6, with little more than twenty-five percent of the total inpatient stays being with a cost higher than €2500. There was also a higher median cost within the accidental falls group compared to the transport injuries group and the self-inflicted injuries group (€1760.4 versus €112.6 and €669.5, resp.). On the whole cost, the medical procedures were significantly proportionally highest for the accidental falls group and for the transport injuries group than for the suicide group (68.0% and 67.3% versus 58%); and the costs of the pharmaceutical procedures were significantly proportionally highest for the suicide group than for the transport injuries group and the accidental falls group (19.5% versus 13.6% and 10.7%, resp.) (Table 11).

## 4. Discussion

To our knowledge, in Belgium, no epidemiological studies have recently investigated specifically the distribution of E codes in the Minimal Clinical Summary database, nor patient characteristics, and even less the cost associated with the hospitalization.

### 4.1. E Codes Observed and Demographic Characteristics of the Patients

As for mortality, transport injuries and self-inflicted injuries were also within the major groups observed [13, 16]. Regarding the gender, more men were present in the transport injuries group but there were more women in the self-inflicted injuries group. This observation is consistent with the known risk factors: women have more attempts on their lives than men [17]. Women were also more present in the accidental falls group. This group is also the one in which patients are older. These two demographic characteristics were equally found in other studies [18, 19].

### 4.2. Moment, Type, and Initiative of the Hospital's Admissions

Our study showed that there were on the whole—and it is the same for the accidental falls group—more admissions on Mondays and Tuesdays than on other weekdays, but there were more admissions for traffic injuries on Saturdays and Sundays and more admissions for suicide and self-inflicted injuries on Sundays and Mondays. It was the same trend as that reported not only by Hawton and van Heeringen [17] in their International Handbook of Suicide and Attempted Suicide, but also by the studies of Beauchamp and colleagues [20] or Colman and colleagues [21].

Regarding the type of admission, there were, for the transport injuries group, more arrivals through the emergency department with the support of a mobile intensive care unit and/or a paramedic intervention team than in the other groups, and relating to the person at the initiative of the admission, the general practitioner was more implicated in the case of an accidental fall. These observations might inform about the seriousness of the traffic injuries, as a mobile intensive care unit's intervention involves “intensive care,” while for accidental falls, the intervention of a general practitioner may be explained by the fact that the patients in this group are older.

### 4.3. Primary Diagnosis That Justifies the Hospitalization

In three-quarters of cases, a diagnosis related to injury and poisoning was reported, with a lower proportion in the accidental falls in which there were more diseases of the circulatory system and in the self-inflicted injuries group, where more mental disorders were found. This difference, in the accidental falls group, must probably be explained by the fact that, in this group, these older patients have more preexisting chronic conditions; the observation in the self-inflicted group is consistent with the literature which reports that there is a significant link between the mental health and the suicidal behaviors [17, 22, 23]. Regarding the injury and poisoning diagnosis group, fractures were the most common diagnosis observed in the accidental falls group and in the traffic injuries group, with a nonnegligible part of intracranial injuries in this last group. These observations were also made in other studies [12, 14, 24]. Finally, in the self-inflicted injuries group, poisoning by drugs and medicinal and biological substances was the most frequent diagnosis observed. This is consistent with the fact that women, who are more represented in this group, were known to choose less violent methods than men, with a predilection for ingesting drugs [22].

### 4.4. Stays Description

A little more than five percent of the whole patients were remaining at least one day in an intensive care unit. In absence of real information about the severity of the injury—as in other European countries, the data do not include, for example, the AIS (abbreviated injury scale)—the seriously injured patients could not be distinguished even if a stay in the intensive care unit is an important indication of the gravity [12]. Regarding the specialized units for the treatment and the rehabilitation, 616 patients have gone through these units after a first ward, with 412 for the musculoskeletal affections. This observation is consistent with the fact that a large number of patients who have had a stay in the specialized units must be linked with the fact that there was an important proportion of limbs fracture. These stays in specialized units might give an idea about the future disabilities of these injured patients, but only a longitudinal follow-up could confirm these disabilities [13, 16].

### 4.5. Type of Discharge, Patient's Destination, and Cause of Death

The proportion of death was higher in the group of accidental falls, with a lower proportion of related death to this fall which has led to the admission. It is certainly correlated with the older age of the patients in this group and with the presence of other diseases as observed in other studies [23]. For the alive patients in this group, a lot of patients returned to their home, and for a little more than one out of ten, the return was toward an elderly home. According to our data we cannot know if the patients were already in this type of structure before their admissions.

In a little less than half of the self-inflicted cases it was the injury and poisoning cause which has been mentioned as the cause of death and; for the alive patients in this group, the proportion of transfer for specialized care and to psychiatric structures as well as of discharge without medical consent was higher than in the other two groups. This need for specific psychiatric care and the problem of unauthorized discharge are clearly documented in suicidology theory [17, 22].

### 4.6. Type, Length of Stay, and Cost Incurred

The median length of stay was nearly equal to one week and was similar to that observed by McKenzie and colleagues [25] in their study. In the accidental falls group, this value is at least three times higher. Scuffham and colleagues [23] have also observed a longer stay for the older elderly patients. Concerning the cost, we have observed that a little more than a quarter of the total inpatient stays have been supported by the social security cost higher than €2500. In the accidental falls group, the median cost was higher than in the other groups; this observation is again correlated with the fact that these patients were older, have other diseases, and have longer hospital stays [12, 13, 23]. Therefore, that is why, on the whole cost, the medical procedures and the day lump sums were proportionally highest in this group. On the other hand, it was the drug cost that was significantly the most important part of the total cost in the suicide group.

Even if it is difficult to compare our costs' results with other studies because of the data's content in our dataset, our observations contributed to the better knowing of the burden of injuries in Belgium. But it is also important to note that taking only into account the hospital costs is (sometimes) only to consider the “tip of the iceberg” because injuries lead (sometimes) to long-term follow-up costs, as shown in the study of Meerding [14] and colleagues who have investigated the costs of injuries based not only on hospital care but also on nursing home care and rehabilitative services.

### 4.7. Quality of the Data

Although there is a ministerial circular that strongly encourages the registration of the codes E when they are present, we have observed that there were shortcomings regarding the encoded information: some cases have only the injury's cause or only the place of occurrence. We have also observed that there is an important utilization of the “.9” code; codes corresponding to the “other accident”/“unspecified cause” and “other place”/“unspecified place” and these codes are generally not useful to researchers because they lack details. These observations were also made in other studies [26–28]. According to McKenzie and colleagues [25] it is essential that clinicians and coders alike be aware of the documentation and coding problems related to the capture of cause-injury data. Another problem is the existence of more than one code of cause. In our study we have elaborated a decision's algorithm, but without knowing the hierarchy of the event, we maybe have made some misinterpretations. In their study, Scuffham and colleagues [23] present the example of a fall occurring on the road which will be coded as traffic injuries. Lawrence and colleagues [27] complete with the fact that “a fall should be E coded only if it causes an injury that is medically treated. If a patient falls down as a result of an illness or poisoning, but does not sustain an injury from the fall, then the fall should not be coded in the patient's record. But we often found records E coded as falls where the only diagnoses are heart conditions.”

In this paper, we have made the choice to not take into account the hospital readmissions. Other authors have made the same choice with the justification that, to consider those events, in a simple way, do not allow overestimation of the impact of injuries [12–14, 18]. Nevertheless, the study of the patients with more than one admission during a year must be really interesting in terms of understanding the repetitive injuries, in terms of investigations of complications/disabilities correlated with the initial injury, and also in terms of incurred costs [18]. It will be also interesting to investigate the costs related to other used health services (e.g., outpatients visits, general practitioner visits, outpatient physical therapy, and home care), as Meerding and colleagues have done [14].

## 5. Conclusion

Our study, the first of such kind in Belgium, has documented the occurrences, the related diagnosis, and the nonnegligible cost for the social security of all types of hospitalized injuries, specifically for three majors groups: the traffic injuries, the suicide and self-inflicted injuries, and finally the accidental falls. In this last group, we have also shown that because these injuries affect the elderly, there is a significant comorbidity which must be also taken into consideration.

Finally, despite the fact that injuries remain an important public health, especially in Belgium, and despite the European Union initiative, our country is not in the list of members' states that have committed to participate to the Joint Action on Monitoring Injuries in Europe (JAMIE) initiative, even it is obvious that the hospital sector provides the best setting for collecting information as this piece of information is related to the more severe cases, and information can be obtained easily on a large number of cases at low cost [5]. The total hospital costs generated by injuries indicate the relative importance of injuries in the healthcare sector as a whole and may be useful in convincing politicians of the importance of preventing injuries and investing in trauma care, and ideally costs and burden of injury should be analyzed in a combined perspective [12, 13].

## Figures and Tables

**Table 1:** Codes of the external causes of injury in the ICD-9-CM taken into account in this study.

	Causes
(E800–E848)	Transport injuries
(E850–E858)	Accidental poisoning by drugs, medicinal substances, and biologicals
(E860–E869)	Accidental poisoning by other solid and liquid substances, gases, and vapors
(E880–E888)	Accidental falls
(E890–E899)	Accidents caused by fire and flames
(E900–E909)	Accidents due to natural and environmental factors
(E910–E915)	Accidents caused by submersion, suffocation, and foreign bodies
(E916–E928)	Other accidents
(E950–E959)	Suicide and self-inflicted injury
(E960–E969)	Homicide and injury purposely inflicted by other persons
(E970–E978)	Legal intervention
(E980–E989)	Injury undetermined whether accidentally or purposely inflicted
(E990–E999)	Injury resulting from operations of war
(E979)	Terrorism
(E929)	Late effects of accidental injury

	Place of occurrence

(E849.0)	Home accidents
(E849.1)	Farm accidents
(E849.2)	Mine and quarry accidents
(E849.3)	Accidents occurring in industrial places and premises
(E849.4)	Accidents occurring in place for recreation and sport
(E849.5)	Street and highway accidents
(E849.6)	Accidents occurring in public building
(E849.7)	Accidents occurring in residential institution
(E849.8)	Accidents occurring in another specified place
(E849.9)	Accidents occurring in unspecified place

**Table 2:** Chapter headings of the ICD-9-CM classification.

(001–139)	Infectious and parasitic diseases
(140–239)	Neoplasms
(240–279)	Endocrine, nutritional, and metabolic diseases and immunity disorders
(280–289)	Diseases of the blood and blood-forming organs
(290–319)	Mental disorders
(320–389)	Diseases of the nervous system and sense organs
(390–459)	Diseases of the circulatory system
(460–519)	Diseases of the respiratory system
(520–579)	Diseases of the digestive system
(580–629)	Diseases of the genitourinary system
(630–676)	Complications of pregnancy, childbirth, and puerperium
(680–709)	Diseases of the skin and subcutaneous tissue
(710–739)	Diseases of the musculoskeletal system and connective tissue
(740–759)	Congenital anomalies
(780–799)	Symptoms, signs, and ill-defined conditions
(800–999)	Injury and poisoning
(V01–V89)	Factors influencing health status and contact with health services

**Table 3:** Subchapter injury and poisoning of the ICD-9-CM classification.

(800–804)	Fracture of skull
(805–809)	Fracture of neck and trunk
(810–819)	Fracture of upper limb
(820–829)	Fracture of lower limb
(830–839)	Dislocation
(840–848)	Sprains and strains of joints and adjacent muscles
(850–854)	Intracranial injury, excluding those with skull fracture
(860–869)	Internal injury of thorax, abdomen, and pelvis
(870–879)	Open wounds of head, neck, and trunk
(880–887)	Open wounds of upper limb
(890–897)	Open wounds of lower limb
(900–904)	Injury to blood vessels
(905–909)	Late effects of injuries, poisonings, toxics effects, and other external causes
(910–919)	Superficial injury
(920–924)	Contusion with intact skin surface
(925–929)	Crushing injury
(930–939)	Effects of foreign body entering through orifice
(940–949)	Burns
(950–957)	Injury to nerves and spinal cord
(958–959)	Certain traumatic complications and unspecified injuries
(960–979)	Poisoning by drugs and medicinal and biological substances
(980–989)	Toxic effects of substances chiefly nonmedicinal as a source
(990–995)	Other and unspecified effects of external causes
(996–999)	Complications of surgical and medical care, not elsewhere classified

**Table 4:** Causes and place of occurrence.

Causes	(*n* = 16406)	Places of occurrence	(*n* = 8493)
(E800–E848)	1841 (11.2)	(E849.0)	3578 (42.1)
(E850–E858)	303 (1.8)	(E849.1)	9 (0.1)
(E860–E869)	191 (1.2)	(E849.2)	4 (<0.1)
(E880–E888)	9555 (58.2)	(E849.3)	167 (2.0)
(E890–E899)	53 (0.3)	(E849.4)	365 (4.3)
(E900–E909)	232 (1.4)	(E849.5)	1140 (13.4)
(E910–E915)	187 (1.1)	(E849.6)	290 (3.4)
(E916–E928)	1474 (9.0)	(E849.7)	1263 (14.9)
(E950–E959)	1745 (10.6)	(E849.8)	199 (2.3)
(E960–E969)	448 (2.7)	(E849.9)	1478 (17.4)
(E970–E978)	1 (<0.1)		
(E980–E989)	235 (1.4)		
(E990–E999)	1 (<0.1)		
(E979)	1 (<0.1)		
(E929)	139 (0.9)		

Data are *n* (%).

**Table 5:** Gender and age of the patients for all injuries and according to the 3 major E codes' groups.

	All injuries	Transport	Falls	Suicide	*P* value
*Gender *	*n* = 17370	*n* = 1841	*n* = 9555	*n* = 1745	<0.001
Male	8305 (47.8)	1228 (66.7)	3756 (39.3)	645 (37.0)	
Female	9065 (52.2)	613 (33.3)	5799 (60.7)	1100 (63.0)	

*Age* (years)	57 (33–80)	32 (20–48)	74 (52–84)	41 (28–50)	<0.001

Data are *n* (%) and median (p25–p75).

**Table 6:** Information about admissions for all injuries and according to the 3 E codes' groups.

	All injuries	Transport	Falls	Suicide	*P* value
*Type of admission, through *	*n = *17370	*n = *1841	*n = *9555	*n = *1745	<0.001
ED, without ambulance	7711 (44.4)	554 (30.1)	4059 (42.5)	609 (34.9)	
ED, with ambulance	6705 (38.6)	587 (31.9)	4353 (45.6)	715 (41.0)	
ED, with ambulance and MICU and/or PIT	2645 (15.2)	670 (36.4)	988 (10.3)	410 (23.5)	
Emergency hospitalization	309 (1.8)	30 (1.6)	155 (1.6)	11 (0.6)	

*At the initiative of *	*n = *17340	*n = *1838	*n = *9539	*n = *1745	<0.001
His/her own initiative	7223 (41.7)	788 (42.9)	3760 (39.4)	791 (45.4)	
A specialist	1093 (6.3)	113 (6.2)	589 (6.2)	40 (2.3)	
His/her insurer	26 (0.2)	4 (0.2)	12 (0.1)	5 (0.3)	
A third party	5430 (31.3)	871 (47.4)	2664 (27.9)	754 (43.3)	
His/her general practitioner	2960 (17.1)	45 (2.5)	2087 (21.9)	122 (7.0)	
A doctor on call	608 (3.5)	17 (0.9)	427 (4.5)	31 (1.8)	

ED: emergency department, MICU: mobile intensive care unit, and PIT: paramedic intervention unit.

**Table 7:** Primary diagnosis (ICD-9-CM) for all injuries and according to the 3 major E codes' groups.

	All injuries	Transport	Falls	Suicide	*P* value
*Primary diagnosis *	*n* = 17370	*n* = 1841	*n* = 9555	*n* = 1745	
(001–139)	153 (0.9)	1 (<0.1)	91 (1.0)	2 (0.1)	
(140–239)	123 (0.7)	—	75 (0.8)	2 (0.1)	
(240–279)	211 (1.2)	1 (<0.1)	144 (1.5)	1 (<0.1)	
(280–289)	52 (0.3)	—	33 (0.4)	—	
(290–319)	576 (3.3)	13 (0.7)	315 (3.3)	126 (7.2)	
(320–389)	440 (2.5)	15 (0.8)	312 (3.3)	7 (0.4)	
(390–459)	870 (5.0)	15 (0.8)	574 (6.0)	1 (<0.1)	
(460–519)	476 (2.7)	5 (0.3)	230 (2.4)	6 (0.3)	
(520–579)	280 (1.6)	1 (<0.1)	129 (1.4)	4 (0.2)	
(580–629)	209 (1.2)	1 (<0.1)	114 (1.2)	3 (0.2)	
(630–676)	23 (0.1)	5 (0.3)	8 (<0.1)	1 (<0.1)	
(680–709)	87 (0.5)	4 (0.2)	22 (0.2)	1 (<0.1)	
(710–739)	379 (2.2)	21 (1.1)	277 (2.9)	2 (0.1)	
(740–759)	3 (<0.1)	—	3 (<0.1)	—	
(780–799)	361 (2.1)	18 (1.0)	216 (2.3)	10 (0.6)	
(800–999)	12955 (74.6)	1714 (93.1)	6903 (72.2)	1570 (90.0)	<0.001*
(V01–V89)	172 (1.0)	27 (1.5)	109 (1.1)	9 (0.5)	

Data are *n* (%). *P value from a dichotomous comparison of (800–999) versus all the other primary diagnoses.

**Table 8:** Injury and poisoning diagnosis (ICD-9-CM) for all stays and according to the 3 major E codes' groups.

	All injuries	Transport	Falls	Suicide
*Injury and poisoning *	*n* = 12955	*n* = 1714	*n* = 6903	*n* = 1570
(800–804)	426 (3.3)	89 (5.2)	223 (3.2)	9 (0.6)
(805–809)	1058 (8.2)	304 (17.7)	703 (10.2)	2 (0.1)
(810–819)	2156 (16.6)	293 (17.1)	1577 (22.9)	2 (0.1)
(820–829)	2949 (22.8)	246 (14.4)	2491 (36.1)	6 (0.4)
(830–839)	293 (2.3)	54 (3.2)	184 (2.7)	2 (0.1)
(840–848)	226 (1.7)	33 (1.9)	101 (1.5)	—
(850–854)	1185 (9.2)	311 (18.1)	693 (10.0)	5 (0.3)
(860–869)	243 (1.9)	100 (5.8)	88 (1.3)	7 (0.5)
(870–879)	313 (2.4)	57 (3.3)	165 (2.4)	11 (0.7)
(880–887)	593 (4.6)	30 (1.8)	40 (0.6)	53 (3.4)
(890–897)	128 (1.0)	31 (1.8)	37 (0.5)	1 (<0.1)
(900–904)	19 (0.2)	2 (0.1)	1 (<0.1)	7 (0.5)
(905–909)	3 (<0.1)	—	3 (<0.1)	—
(910–919)	42 (0.3)	6 (0.4)	5 (<0.1)	2 (0.1)
(920–924)	528 (4.1)	125 (7.3)	294 (4.3)	7 (0.5)
(925–929)	36 (0.3)	5 (0.3)	4 (<0.1)	—
(930–939)	65 (0.5)	—	1 (<0.1)	—
(940–949)	173 (1.3)	2 (0.1)	5 (<0.1)	3 (0.2)
(950–957)	41 (0.3)	2 (0.1)	11 (0.2)	4 (0.3)
(958–959)	201 (1.6)	19 (1.1)	138 (2.0)	8 (0.5)
(960–979)	1644 (12.7)	—	23 (0.3)	1321 (84.1)
(980–989)	309 (2.4)	1 (<0.1)	14 (0.2)	108 (6.9)
(990–995)	125 (1.0)	2 (0.1)	15 (0.2)	12 (0.8)
(996–999)	199 (1.5)	2 (0.1)	87 (1.3)	—

Data are *n* (%).

**Table 9:** Vital status, discharge, and destination for all injuries and according to the 3 major E codes' groups.

	All injuries	Transport	Falls	Suicide	*P* value
*Vital status *	*n* = 17349	*n* = 1839	*n* = 9544	*n* = 1741	<0.001
Death	726 (4.2)	46 (2.5)	490 (5.1)	22 (1.3)	

*Type of discharge *	*n* = 16623	*n* = 1793	*n* = 9054	*n* = 1719	<0.001
On medical advice	15664 (94.2)	1692 (94.4)	8605 (95.0)	1469 (85.5)	
Without consent	315 (1.9)	22 (1.2)	87 (1.0)	138 (8.0)	
Transfer for specialized care	448 (2.7)	65 (3.6)	214 (2.4)	100 (5.8)	
Transfer for reeducation	167 (1.0)	17 (0.8)	126 (1.4)	9 (0.5)	
Transfer for logistic reasons	29 (0.2)	0 (0.0)	22 (0.2)	3 (0.2)	

*Type of destination *	*n* = 16603	*n* = 1790	*n* = 9050	*n* = 1714	<0.001
Domicile	14271 (86.0)	1664 (93.0)	7345 (81.2)	1555 (90.7)	
Another hospital	534 (3.2)	92 (5.1)	329 (3.6)	32 (1.9)	
Elderly homes	1452 (8.8)	13 (0.7)	1254 (13.9)	12 (0.7)	
Psychiatric homes/hospitals	207 (1.2)	2 (0.1)	59 (0.6)	98 (5.7)	
Others	139 (0.8)	19 (1.1)	63 (0.7)	17 (1.0)	

Data are *n* (%).

**Table 10:** Death's causes for all injuries and according to the 3 major E codes' groups.

	All injuries	Transport	Falls	Suicide
*Cause of death *	*n* = 573*	*n* = 38	*n* = 384	*n* = 17
(E800–E848)	12 (2.1)	12 (31.6)	—	—
(E850–E858)	1 (0.2)	—	—	—
(E878–E879)	1 (0.2)	—	1 (0.3)	—
(E880–E888)	68 (11.9)	—	67 (17.5)	—
(E890–E899)	2 (0.4)	—	1 (0.3)	—
(E910–E915)	7 (1.2)	—	1 (0.3)	—
(E950–E959)	5 (0.9)	—	—	5 (29.4)
(001–139)	15 (2.6)	—	8 (2.1)	—
(140–239)	66 (11.5)	—	47 (12.2)	1 (5.9)
(240–279)	5 (0.9)	—	4 (1.0)	—
(280–289)	1 (0.2)	—	1 (0.3)	—
(290–319)	3 (0.5)	—	3 (0.8)	—
(320–389)	11 (1.9)	1 (2.6)	4 (1.0)	1 (5.9)
(390–459)	156 (27.2)	14 (36.8)	106 (27.6)	1 (5.9)
(460–519)	64 (11.1)	—	42 (10.9)	—
(520–579)	26 (4.5)	—	16 (4.2)	—
(580–629)	9 (1.6)	—	6 (1.6)	—
(630–676)	0 (0.0)	—	—	—
(680–709)	1 (0.2)	—	1 (0.3)	—
(710–739)	3 (0.5)	—	2 (0.5)	—
(780–799)	35 (6.1)	2 (5.3)	26 (6.8)	2 (11.8)
(800–999)	82 (14.3)	9 (23.7)	48 (12.5)	7 (41.2)

Data are *n* (%). *In the 726 deaths, there were 153 missing causes.

**Table 11:** Type, length, and cost of stays for all stays and according to the 3 major E codes' groups.

	Total	Transport	Falls	Suicide	*P* value
*Type of stay *	*n* = 17370	*n* = 1841	*n* = 9555	*n* = 1745	<0.001
Inpatient	15626 (90.0)	1680 (91.3)	8825 (92.4)	1653 (94.7)	
Day surgery	528 (3.0)	18 (1.0)	165 (1.7)	6 (0.3)	
Day hospitalisation	1097 (6.3)	136 (7.4)	515 (5.4)	84 (4.8)	
Outpatient emergency	119 (0.7)	7 (0.4)	50 (0.5)	2 (0.1)	

Length of stay*	*n* = 15626	*n* = 1680	*n* = 8825	*n* = 1653	<0.001
Stay (days)	6 (2–15)	3 (2–8)	9 (3–19)	2 (2–5)	

*Cost *(*euros*)*	*n* = 15174	*n* = 1617	*n* = 8619	*n* = 1616	
Total cost	1376.6 (751.4–2584.5)	1112.6 (698.8–2091.8)	1760.4 (1006.8–3049.6)	669.5 (564.3–1031.9)	<0.001
Medical procedures	949.6 (449.4–1704.4)	777.4 (414.7–1456.7)	1210.4 (665.6–1958.7)	382.3 (298.4–685.9)	<0.001
Pharmaceutical products	150.3 (129.2–200.3)	141.3 (126.0–180.5)	160.0 (132.5–217.1)	130.0 (120.6–150.2)	<0.001
Day lump sums	192.7 (139.0–315.7)	167.2 (123.2–236.0)	212.1 (145.7–353.1)	157.1 (120.8–198.7)	<0.001

Percentages of the total cost*	*n* = 15174	*n* = 1617	*n* = 8619	*n* = 1616	
Medical procedures	66.1 (55.5–74.7)	67.3 (55.6–75.9)	68.0 (57.9–75.6)	58.0 (52.0–67.3)	
Pharmaceutical products	12.5 (7.9–19.2)	13.6 (8.7–19.7)	10.7 (7.0–16.2)	19.5 (13.9–23.3)	
Day lump sums	16.1 (9.8–23.1)	15.3 (8.8–23.1)	14.4 (8.9–20.9)	22.1 (16.4–26.9)	

*Only for the inpatients. Data are *n* (%) and median (p25–p75).
